# Reproducibility of daytime hypertension, night-time hypertension, and nocturnal blood pressure dipping patterns in young to middle age patients with stage 1 hypertension

**DOI:** 10.1097/HJH.0000000000003874

**Published:** 2024-10-11

**Authors:** Paolo Palatini, Francesca Battista, Lucio Mos, Marcello Rattazzi, Andrea Ermolao, Olga Vriz, Adriano Mazzer, Francesca Saladini

**Affiliations:** a Department of Medicine - University of Padova, Padova; b San Antonio Hospital, San Daniele del Friuli; c Vittorio Veneto Town Hospital, Vittorio Veneto; d Cittadella Town Hospital, Cittadella, Italy

**Keywords:** ambulatory, blood pressure, daytime, night-time, nocturnal hypertension, non dipper, reproducibility

## Abstract

**Objective:**

To investigate the reproducibility of ambulatory BP sub-periods and nocturnal dipping phenotypes assessed twice 3 months apart in young-to-middle-age untreated individuals screened for stage 1 hypertension.

**Design and methods:**

We investigated 1096, 18-to-45-year old participants from the HARVEST. Their office BP was 145.8 ± 10.4/93.7 ± 5.7 mmHg. Office BP and 24 h BP were measured at baseline and after 3 months. Office, 24-h, daytime and night-time hypertensions, and nocturnal dipping patterns were defined according to the 2023 ESH guidelines. Between-recording agreement was evaluated with kappa statistics.

**Results:**

Reproducibility evaluated with weighted kappa was moderate for both 24 h hypertension (*K* = 0.48) and daytime hypertension (*K* = 0.50) and was only fair for night-time hypertension (*K* = 0.36). Between-measurement agreement was even worse for isolated night-time hypertension (*K* = 0.24), and was poor for office hypertension (*K* = 0.14). The better reproducibility of daytime than night-time period was confirmed by the analysis of BP as continuous variable (all between-period differences, *P* < 0.001). Nondipping was present in 31.8%, and showed a fair agreement (*K* = 0.28,). Poorer agreement was shown by extreme dipping (*K* = 0.18) and reverse dipping (*K* = 0.07).

**Conclusions:**

These data show that within the ambulatory sub-periods, daytime hypertension has a better reproducibility than night-time hypertension. This suggests that the better association with adverse outcomes shown by sleep BP compared to wake BP in observational studies is not due to a better reproducibility of the former. The between-measurement agreement is even worse for isolated nocturnal hypertension and dipping patterns, especially for extreme and reverse dipping. Thus, these BP phenotypes should be confirmed with repeat ambulatory BP monitoring.

## INTRODUCTION

A body of evidence has demonstrated that ambulatory blood pressure (BP) has a greater predictive value for cardiovascular events and mortality than office BP [1–3]. In addition, it has been shown that within the ambulatory 24-h (ABPM) period BP measured during sleep has a stronger association with adverse outcomes than BP during daylight hours. The greater prognostic significance of night-time BP has been observed in general populations and several clinical conditions such as chronic kidney disease or diabetes [4–6]. Overall, the presence of high BP at night seems to be more harmful than hypertension during daytime hours [7]. The reason for the prognostic superiority of nocturnal over diurnal BP is not obvious and several possible explanations have been suggested. Among these, the more standardized conditions during sleep (supine position and absence of activities), which would translate into a better BP reproducibility, were considered the main reason for the closer association of night-time BP with adverse cardiovascular outcomes than daytime BP [4–7].

The better reproducibility of ambulatory BP compared to office BP has been known for long [1,8]. However, previous research focused especially on the reproducibility of white-coat and masked hypertension [9,10] and only did a few studies evaluate the reproducibility of ABPM subperiods and of 24-h BP rhythm patterns [11]. In addition the between-measurement agreement was generally evaluated from the width of the 95% limits of agreement of intraindividual BP [11] and only did a few studies focus on the reproducibility of ambulatory hypertension phenotypes and of extreme and reverse dipping. In addition, most data were obtained from small samples and one third from hypertensive patients on antihypertensive treatment [11].

Thus, the purpose of the present study was to investigate the short term reproducibility of ambulatory hypertension phenotypes and several indexes of diurnal BP rhythm with the main aim of comparing the agreement of night-time versus daytime hypertension. These data were obtained from the largest longitudinal study which performed two ABPMs at baseline within a short period of time in a cohort of untreated patients with hypertension.

## METHODS

### Subjects

The analysis was carried out in 1096 white subjects (797 men) who took part in the HARVEST, a study on the predictive value of office versus ambulatory BP for the development of sustained hypertension and cardiovascular events in young-to-middle-age patients screened for stage 1 hypertension [12–14]. The HARVEST is conducted in 17 hypertension units in Italy beginning on April 1990 [12–14]. Subjects with office systolic BP (SBP) from 140 to 159 mmHg and/or diastolic BP (DBP) from 90 to 99 mmHg were enrolled. Their mean age was 33.0 ± 8.6 years, and their body weight 76.3 ± 13 kg. None of the participants had ever taken antihypertensive therapy. Subjects with diabetes, nephropathy, cardiovascular disease, neoplastic diseases and any other serious clinical condition were excluded. More details on the procedures used in the HARVEST, including the evaluation of lifestyle factors (smoking, alcohol drinking, coffee use, and physical activity habits), can be found elsewhere [12–14]. The protocol was approved by the Ethics Committee of the HARVEST [14] and the procedures were performed in accordance with the ethical standards as laid down in the 1964 Declaration of Helsinki and its later amendments. All subjects gave written informed consent. A total of 1096 participants in whom office and 24-h BP were available at baseline and after three months of observation and who did not receive any antihypertensive treatment were considered.

### BP measurement

Office BP was measured three times after subjects had rested 5 min in the supine position. The mean of the three readings was defined as office BP. Standard (12 × 24 cm) and large (15 × 30 cm) cuffs were used according to participants’ arm size for both office and ambulatory BP measurements. Office BP was measured just before the application of the ABPM device. ABPMs were obtained with the A&D TM-2420 model 7 or with the ICR Spacelabs 90207. Both devices were previously validated and were shown to provide comparable results [15,16]. Before starting the study all investigators underwent common training procedures on the use of the recorders to ensure methodological homogeneity. Before the recording was started, the device was checked against a mercury sphygmomanometer by means of a Y tube. Six subsequent measurements provided by the recorder, three in the lying and three in the standing position, were required to agree (±5 mmHg) with simultaneous ambulatory measurements taken by the doctor. When the device was in operation, participants were requested to keep their arm still and to remain motionless. They were invited to follow their ordinary daily routine during the recording and as far as possible to perform the same pattern of activity during the repeat ABPM. They were asked to go to bed not later than 11 p.m. BP was measured every 10–15 min during waking hours (6 a.m. to 11 p.m.) and every 30 min during sleep [17]. Participants were instructed to go to bed and to wake up according to our scheduled times. Patient's adherence was checked from the diary card. After 3 months, ABPM was repeated following the same procedures used at baseline.

### ABPM data analysis

All ABPM data were sent to the Coordinating Office in Padova where they were screened for editing of artifactual values based on previously described criteria [13]. Only recordings containing error measurements of 20% or less were considered acceptable for evaluation. A total of 9.3% of the ABPMs were rejected because the artifactual BP readings exceeded 20% of the measurements. The arithmetic average of the edited pressures was used as the ambulatory measurement for each recording period. Average 24-h BP, daytime BP (6 .m. to 11 p.m.), night-time BP (11 p.m. to 6 a.m.), and day-night BP difference were calculated. Body mass index (BMI) was calculated as the ratio of body weight to squared height.

### Definitions

Participants were categorized according to the level of office and average 24-h, daytime and night-time SBP and/or DBP measured at baseline and after 3 months. The cutoff values used to define normal and high BPs were 140/90 mmHg for office BP, 130/80 mmHg for 24-h BP, 135/85 mmHg for daytime BP and 120/70 mmHg for night-time BP [18]. Isolated nocturnal hypertension was defined as asleep BP ≥120/70 mmHg with awake BP < 135/85 mmHg [18].

### Nocturnal BP fall

We calculated the night–day BP ratio as the ratio of average night-time BP to average daytime BP. Using the ambulatory BP nocturnal decline in percentage we identified four groups according to standard criteria [17,18]: dippers (>10–20%), nondippers (>0–10%), reverse dippers (≤0%), and extreme dippers (>20%).

### Statistics

Baseline and repeat periods of recording were compared with the Student's *t* test for paired observations. The other comparisons were made by means of the Student's *t* test for unpaired observations or the analysis of variance and Tukey's post hoc test where indicated. Categorical variables were analysed with the chi-square test. For correlations the Pearson test was used. Predictors of between-ABPM BP changes were evaluated in a linear multivariable regression analysis, and predictors of the reproducibility of daytime and night-time hypertension in a multivariable logistic regression. The reproducibility of hypertension phenotypes was evaluated using kappa statistics, which measure agreement occurring in excess of that expected by chance. Weighted kappa (*K*) was calculated according to the Cohen's method using linear weight [19]. The standard error and 95% confidence interval were calculated according to Fleiss *et al.*[20]. According to Altman, the strength of agreement can be defined as poor if kappa is <0.20, fair if kappa is 0.21–0.40, moderate if kappa is 0.41–0.60, good if kappa is 0.61–0.80, and very good if kappa is 0.81–1.00 [21]. Between-measurement agreement was tested also using the intraclass correlation approach [22]. Reproducibility of BP considered as a continuous variable was assessed using correlation coefficient, coefficient of variation, within-subject standard deviation, and Bland-Altman approach [21]. Change between duplicate recordings was calculated by subtracting the first from the second recording, taking into account the sign of the difference. Significance was taken as a probability value less than 0.05.

## RESULTS

The main clinical characteristics of the 1096 participants are reported in Table S1, Supplemental Digital Content. Their office BP was in the stage 1 hypertensive range. Due to the natural selection of people with high BP in the 18–45 year age range, there was a higher prevalence of males (72.7%). Average ambulatory 24-h BP was 14.8/12.4 mmHg lower than office BP. The average numbers of awake and asleep BP readings were 72.1 ± 19.4 and 13.3 ± 1.5, respectively.

After three months of follow-up, mean office BP fell by 5.3/3.2 mmHg, while only a small decline was observed for 24-h and daytime BPs (Table 1). Night-time BP showed a negligible nonsignificant increase at repeat ABPM. Body weight declined by 0.48 ± 3.0 kg (*P* < 0.001).

**TABLE 1:** Office and ambulatory blood pressures measured at baseline (1st measurement) and after 3 months (second measurement)

			Paired differences			
Variable	1st measurement		2nd measurement				*P*-value	
	Mean	SD	Mean	SD	Mean	SD	Uncorrected	Corrected^∗^
Office systolic BP	145.8	10.4	140.5	12.1	−5.26	11.8	<0.001	<0.001
Office diastolic BP	93.7	5.7	90.5	8.5	−3.23	7.9	<0.001	<0.001
24-h systolic BP	131.0	10.8	130.6	11.0	−0.44	8.1	0.07	0.56
24-h diastolic BP	81.3	8.2	80.9	8.4	−0.44	6.3	0.020	0.16
Daytime systolic BP	134.6	11.2	133.7	11.4	−0.82	8.6	0.002	0.016
Daytime diastolic BP	83.7	8.6	83.0	8.8	−0.70	6.7	0.0006	0.005
Night-time systolic BP	117.4	11.8	117.5	12.3	0.18	10.3	0.56	1.0
Night-time diastolic BP	72.3	8.7	72.6	8.9	0.36	7.8	0.12	0.96

*P*-values from paired sample *t*-test.

∗ With Bonferroni correction for multiple comparisons. BP indicates blood pressure in mmHg.

### Categorical data

**FIGURE 1:**
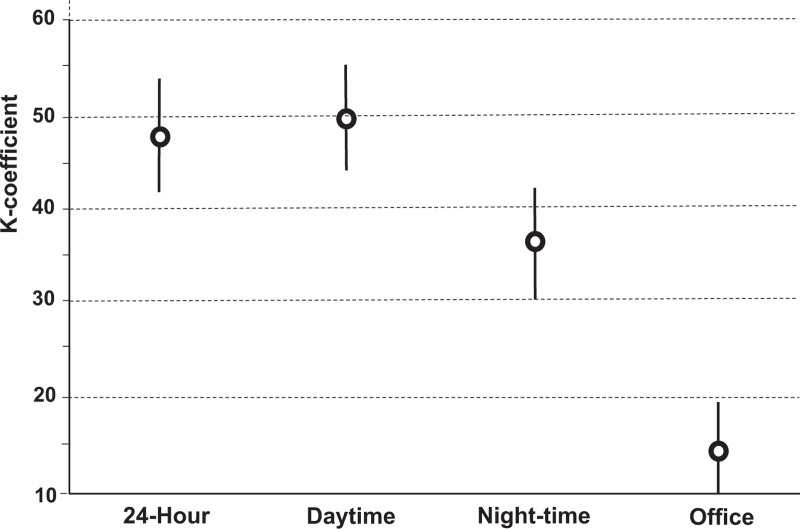
Reproducibility of office and ambulatory hypertensions diagnosed at baseline and after 3 months of observation in 1096 HARVEST participants. The figure shows weighted kappa coefficients and 95% confidence interval calculated according to the Cohen's method.

**FIGURE 2:**
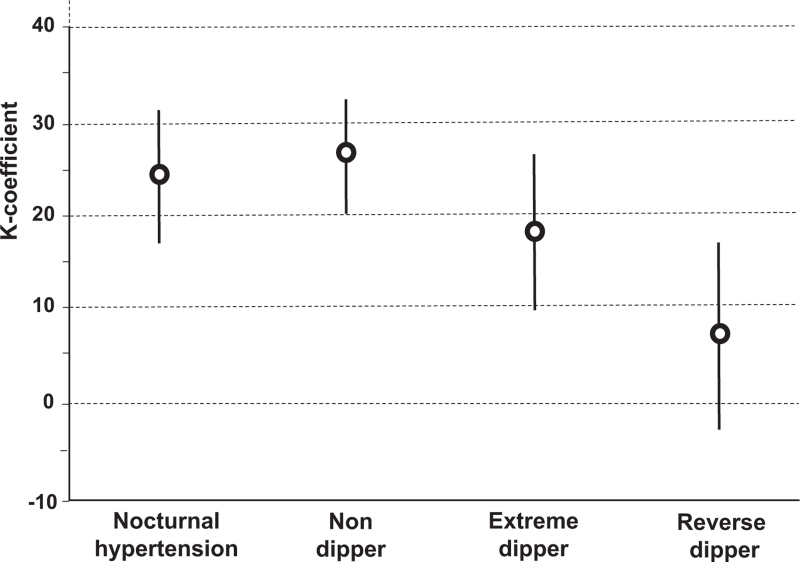
Reproducibility of isolated nocturnal hypertensions and of dipping phenotypes diagnosed at baseline and after 3 months of observation in 1096 HARVEST participants. The figure shows weighted kappa coefficients and 95% confidence interval calculated according to the Cohen's method.

At baseline, 77.4% of the participants showed 24-h hypertension, 67.7% daytime hypertension, and 70.0% night-time hypertension. Hypertension reproducibility over the 3-month period, evaluated with weighted kappa, was moderate for average 24-h and daytime BPs and only fair for night-time BP (Fig. 1). No overlap was observed between the confidence intervals of daytime and night-time *K*-coefficients which is consistent with a statistically significant difference between the two ambulatory subperiods. For night-time but not daytime hypertension better agreement was found in women than men (Table S2, Supplemental Digital Content). Isolated nocturnal hypertension was present at baseline in 13.8% of the participants but was confirmed in only 28.9%, with even smaller *K*-coefficient than night-time hypertension without differences between the two sexes (Fig. 2 and Table S2, Supplemental Digital Content). Office hypertension showed a poor agreement with weighted *K*-coefficient well below those of all ambulatory hypertensions. Similar results were obtained using the intraclass correlation approach, which also documented a better agreement for 24-h and daytime hypertensions compared with night-time hypertension (Table S3, Supplemental Digital Content).

### Reproducibility of dipping status

Non dipping was present in 31.8% of the participants at baseline and was confirmed in 59.8%. At kappa statistics, it showed a fair agreement (Fig. 2) with slightly higher *K*-coefficient in women than men (Table S2, Supplemental Digital Content). The agreement was worse for extreme dipping (9.4% rate) and reverse dipping (3.2% rate), which were confirmed in only 29.8% and 8.3% of participants, respectively (Fig. 2). When the diurnal rhythm data were analysed with the intraclass correlation test, consistent results were obtained (Table S3, Supplemental Digital Content).

### Continuous variables

**FIGURE 3:**
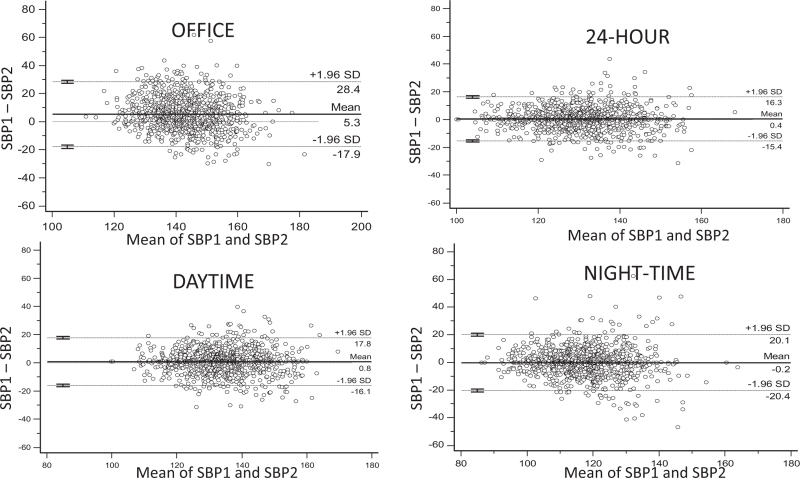
Reproducibility of office and ambulatory systolic blood pressures measured at baseline and after 3 months of observation in 1096 HARVEST participants. The figure shows the Bland-Altman plots with 95% limits of agreement (dashed lines). The solid line represents the mean difference between the two measurements. SBP1 indicates systolic blood pressure at baseline in mmHg; SBP2, systolic blood pressure at repeat recording in mmHg.

Correlation coefficients between baseline and repeat office and ambulatory BP measurements are shown in Table S4, Supplemental Digital Content. For both SBP and DBP, correlation coefficients were higher for 24-h and daytime BPs than for night-time BP (*P* ≤ 0.001). The coefficient of variation and within-subject standard deviation were also higher for 24-h and awake BPs than for sleep BP (*P* < 0.001) (Table 2). These results were confirmed by the analysis of the Bland-Altman plots which showed better agreement for daytime than night-time BP, as documented by the narrower confidence interval of both systolic (Fig. 3) and diastolic between-monitoring BP differences. In all analyses, office BP showed a poorer reproducibility than ambulatory BPs.

**TABLE 2:** Mean blood pressure, coefficient of variation, and within-subject standard deviation for office and ambulatory blood pressures measured twice 3 months apart

Variable	Overall mean	Coefficient of variation	Within-subject SD
Office systolic BP, mmHg	143.17	6.38	9.13
Office diastolic BP, mmHg	92.13	6.56	6.04
24-h systolic BP, mmHg	130.84	4.77	5.71
24-h diastolic BP, mmHg	81.15	5.55	4.50
Daytime systolic BP, mmHg	134.19	4.57^∗^^,^^†^	6.14^∗^^,^^†^
Daytime diastolic BP, mmHg	83.42	5.75^∗^^,^^†^	4.80^∗^^,^^†^
Night-time systolic BP, mmHg	117.49	6.21	7.30
Night-time diastolic BP, mmHg	72.48	7.65	5.54

BP, blood pressure; SD, standard deviation.

∗ *P* < 0.0001 versus night-time BP.

† *P* = N.S. versus 24-h BP (from variance ratio test).

### Factors affecting hypertension reproducibility

In a logistic regression, we investigated the association between several clinical variables and the consistency in the diagnosis of daytime and night-time hypertension (dependent variables). Age, sex, BMI, ambulatory SBP and DBP, ambulatory heart rate, standard deviation of the mean SBP and DBP (as a measure of BP variability), smoking, alcohol drinking, coffee use, physical activity habits, BP reaction to standing, and number of valid BP readings were used as independent variables. The level of night-time SBP and DBP (*P* < 0.001 for both), and BMI (*P* = 0.038) were predictors of night-time hypertension reproducibility. The level of daytime SBP and DBP (*P* < 0.001 for both), and smoking (in an inverse fashion, *P* = 0.006) were associated with daytime hypertension reproducibility. The number of BP readings was similar in the participants with consistent and inconsistent diagnosis of night-time hypertension (13.4 ± 1.5 versus 13.3 ± 1.5 readings, respectively, *P* = 0.88) or daytime hypertension (73.0 ± 19.1 versus 71.5 ± 19.5 readings, respectively, *P* = 0.21).

### Factors affecting between-measurement BP changes

The only consistent predictor of daytime and night-time SBP and DBP changes was the level of ambulatory BPs (*P* < 0.001 for all). The higher the participant's BP, the higher the probability of having a greater difference (positive or negative) at repeat recording. Male sex was a significant predictor of both daytime (*P* = 0.001) and night-time SBP (*P* = 0.019) changes, whereas age was a predictor of daytime and night-time DBP differences (both *P* < 0.001). Baseline BMI (*P* = 0.007) and change in body weight from 1st to 2nd measurement (*P* = 0.006) were associated with changes in daytime BP but not in night-time BP. Smoking, alcohol drinking, coffee use, physical activity habits, ambulatory heart rate, BP variability, and the BP response to standing were not associated with ambulatory BP changes from baseline to repeat recording.

## DISCUSSION

The present results confirm that ABPM has an excellent population-based short-term reproducibility as only negligible changes in mean daytime and night-time SBP and DBP were found from the first to the second ABPM (all <1 mmHg). However, the intra-individual reproducibility was limited especially for some ABPM indexes. Nocturnal hypertension showed only a fair agreement when reassessed after 3 months, and a lower reproducibility than both 24-h and daytime hypertensions. The agreement was even lower for isolated nocturnal hypertension. In addition, our study confirmed that intraindividual classification of dippers and nondippers was unstable as around 40% of participants had their dipping status changed on repeated measurements. The agreement was particularly poor for extreme dipping and reverse dipping.

### Reproducibility of daytime versus night-time hypertension

In ABPM studies nocturnal hypertension has been found to be a stronger predictor of cardiovascular events compared with 24-h or daytime hypertension [4–7,23]. Recent data from the Spanish ABPM registry showed that night-time SBP was 6 times more predictive of death than SBP assessed with standard office measurement [6]. Possible explanations for the strong predictive value of night-time hypertension are the better prognostic ability of BP measured in sedated condition [24] and the absence of confounding factors unrelated to cardiovascular risk during sleep which allows BP measurement in more standardized conditions [7]. According to most investigators this would lead to a better reproducibility of BP measured during sleep than during waking hours. However, this hypothesis was not supported by the present findings. According to kappa statistics, night-time hypertension showed only a fair agreement when evaluated twice three months apart (*K*-coefficient, 0.37) and a lower reproducibility than daytime hypertension (*K*-coefficient, 0.50). These results were confirmed by the intraclass correlation coefficient statistical approach. The reproducibility of sleep BP was particularly low in the male participants (*K*-coefficient, 0.33). This may account for the lower predictive value of night-time BP for cardiovascular events in in men than women found in a meta-analysis of 11 populations [25]. In the general population, up to 20% of individuals may exhibit isolated nocturnal hypertension [26,27] a condition associated with increased cardiovascular risk [23,28]. However, in agreement with previous reports [29] the reproducibility of this hypertension phenotype, present in 13% of our participants, was low with a K-coefficient of 0.24.

The better reproducibility of daytime compared to night-time hypertension was not due to the arbitrary cut-offs which were used to define these conditions. Also when ambulatory BP was considered as a continuous variable, daytime SBP and DBP showed a better agreement over the 3-month period than night-time SBP and DBP as documented by the higher coefficients of variation, and within-subject standard deviations. In addition, Bland-Altman plots showed narrower limits of agreement for BP measured during wake than sleep. The results of this statistical approach confirm the results of a recent meta-analysis of six studies [11] in which narrower 95% limits of agreement were found for daytime (−16.7–18.4 mmHg for SBP, and −10.4–12.3 mmHg for DBP) than for night-time BP (−19.6–21.3 mmHg for SBP, and −11.3–12.4mmHg for DBP). However, some studies based on smaller samples reported a better reproducibility of asleep than awake BP, although a different statistical approach was used (Correlation coefficients and Standard deviations of the between-monitoring differences) [30].

### Factors affecting reproducibility

The exact reasons for the limited reproducibility of night-time BP and night-time hypertension are unclear. Daytime and night-time BP can be influenced by several confounding factors [31–36]. In our study, the main determinants of both night-time and daytime hypertension reproducibility were the level of ambulatory SBP and DBP. BMI for night-time hypertension, and smoking (in an inverse fashion) for daytime hypertension were other factors associated with hypertension consistency. When BP changes from one day to the other were considered as the dependent variables, age, male sex, and the level of BP were found to be independently associated with both sleep and waking BP changes. Changes in body weight over the 3 months increased daytime but not night-time between-monitoring variability. A number of other unmeasured factors, such as season temperature [31,32] and lifestyle factors [37] can increase the between-recording variability. Quality of sleep and episodes of sleep apnoea may vary from one recording to the other thereby affecting the level of nocturnal BP during repeated measurements [34,35]. An improvement of sleep quality during the second recording could account for the intraindividual variability of sleep BP. However, night-time BP was not lower at repeat ABPM and it is thus unlikely that the poor sleep BP reproducibility was due to an adaptation to the ABPM procedure at repeat recording. Changes in physical activity and other behavioural activities and in body posture during waking hours can affect mean daytime BP [33]. Smoking, a source of BP variability [38] and a potential confounding factor during waking time was not a determinant of daytime BP changes from day to day but was a negative predictor of daytime hypertension reproducibility. Alcohol drinking, coffee use, and physical activity habits did not influence the reproducibility of ambulatory BP measurements. Other possible factors potentially influencing the level of either daytime or sleep BP such as ambulatory heart rate, short-term BP variability, and the BP response to standing [39] were unrelated to ambulatory BP changes from baseline to repeat recording. The larger number of BP readings during waking hours could have favoured the reproducibility of daytime BP. However, the number of both daytime and night-time BP readings did not differ according to the reproducibility of hypertension phenotypes.

### Diurnal BP rhythm indexes

Nocturnal non dipping (BP fall < 10%) and particularly reverse dipping, have been linked to an increased risk for adverse cardiovascular outcomes [40–42]. Also extreme dipping has been found to be associated with cardiovascular events in elderly individuals [43]. Thus, identification of abnormalities of daily BP rhythms may be helpful for the prevention and management of hypertensive complications. However, the reproducibility of nondipping has been found to be poor with one quarter to one-third of individuals having their dipping status changed on repeated measurements [11,44]. Reproducibility is even worse in patients receiving medication, as shown by the ELSA study [45]. Almost 50% of the participants had a different dipping phenotype at the second measurement with lower rates for extreme dippers and reverse dippers. The present data show that dipping phenotypes have a poor reproducibility also in untreated hypertensive patients. *K*-coefficient for nondipping was 0.27 with poorer agreement of dipping status in men than women. The cardiovascular risk associated with diurnal rhythm abnormalities is particularly strong when night-time BP is higher than daytime BP [46]. However, the reproducibility of this condition is very poor as shown by the low K-coefficient (0.07) in the present study. The agreement was poor also for extreme dipping (nocturnal BP fall > 20%) a condition associated with increased cardiovascular risk in the elderly [43]. These findings reflect the action of several confounding factors that affect both night-time and daytime BPs thereby increasing the instability of parameters that incorporate the variability of both periods.

### Limitations

Several limitations of this study should be acknowledged. First, changes in participants’ lifestyle after enrolment may have influenced BP after three months, as suggested by the slight decrease in body weight. However, changes in body weight were not associated with sleep BP variability and it is thus unlikely that they contributed to the poorer reproducibility of night-time BP. In addition, previous data from the HARVEST showed that only minor changes in participants’ lifestyle habits occurred during the follow-up [37]. Second, we have no information on several factors potentially affecting the between-measurement agreement, such as physical activity and body posture during daily light hours or sleep quality and duration during the night-time. However, to improve the reproducibility of the ambulatory subperiods, a fixed time interval was used during sleep, and care was taken to minimize differences in daylight activities during the second recording. Third, our participants were not selected from a general population but from a population of young-to-middle-age individuals screened for stage 1 hypertension and thus our findings may not be generalized to other populations.

## CONCLUSION

Accumulating evidence has shown that nocturnal hypertension and abnormalities of diurnal BP rhythm are associated with increased cardiovascular risk. Thus, according to current guidelines, the evaluation of night-time BP and nocturnal BP fall is an indication for ABPM in hypertensive patients. However, in keeping with previous data the present results show that nocturnal hypertension and abnormalities of diurnal rhythm are unstable conditions. Nocturnal hypertension, especially when isolated, was confirmed in only one third of the participants. A poor agreement was also shown for dipping phenotypes especially for the categories at the extremes of the nocturnal BP fall distribution. These data indicate that in spite of their better reproducibility compared to office BP, several ABPM phenotypes have a limited stability over time. Therefore, when isolated nocturnal hypertension or abnormalities of the diurnal rhythm are detected the diagnosis should be confirmed with a repeat ABPM.

## ACKNOWLEDGEMENTS

Funding. This study was funded by the Associazione “18 Maggio 1370”, San Daniele del Friuli, Italy.

### Conflicts of interest

There are no conflicts of interest

## Supplementary Material

Supplemental Digital Content
