# Supplementary material for: Reproducibility of daytime hypertension, night-time hypertension, and nocturnal blood pressure dipping patterns in young to middle age patients with stage 1 hypertension
Source: J Hypertens. 2024 Oct 11;43(1):128–35. doi: 10.1097/HJH.0000000000003874 (PMC11608614; doi:10.1097/HJH.0000000000003874)
Supplement: Supplemental Digital Content [file jhype-43-128-s001.docx]

**Table S1.** Clinical characteristics of the study participants.

| **Variable** | **Mean** | **SD** |
| --- | --- | --- |
| Age, years | 33.0 | 8.5 |
| Body mass index, Kg/m^2^ | 25.4 | 3.4 |
| Office systolic BP, mmHg | 145.8 | 10.4 |
| Office diastolic BP, mmHg | 93.7 | 5.7 |
| Office heart rate, bpm | 74.7 | 9.6 |
| 24-hour systolic BP, mmHg | 131.0 | 10.8 |
| 24-hour diastolic BP, mmHg | 81.3 | 8.2 |
| Serum glucose, mg/dL | 92.8 | 11.2 |
| Total cholesterol, mg/dL | 196.7 | 40.06 |
| HDL-cholesterol, mg/dL | 52.7 | 14.5 |
| Triglycerides, mg/dL | 112.7 | 71.6 |
| Sex (men), % | 72.7 | ---- |
| Smokers, % | 20.6 | ---- |
| Alcohol drinkers, % | 47.2 | ---- |

BP indicates blood pressure

**Table S2**. Kappa coefficients for ambulatory blood pressure phenotypes in the participants grouped by sex.

| **Variable** | **Kappa coefficient** | **95% Confidence Interval** |
| --- | --- | --- |
| Daytime hypertension women | 0.484 | 0.383 to 0.583 |
| Daytime hypertension men | 0.503 | 0.439 to 0.566 |
| Night-time hypertension women | 0.429 | 0.321 to 0.537 |
| Night-time hypertension men | 0.330 | 0.256 to 0.403 |
| Isolated nocturnal hypertension women | 0.214 | 0.084 to 0.342 |
| Isolated nocturnal hypertension men | 0.247 | 0.165 to 0.328 |
| Non dipper women | 0.304 | 0.186 to 0.421 |
| Non dipper men | 0.250 | 0.179 to 0.320 |

**Table S3.** Intraclass correlation coefficients for ambulatory blood pressure phenotypes.

| **Variable** | **Intraclass correlation** | **95% Confidence Interval** |
| --- | --- | --- |
| 24-hour hypertension | 0.477 | 0.430 to 0.522 |
| Daytime hypertension | 0.504 | 0.458 to 0.546 |
| Night-time hypertension | 0.366 | 0.3141to 0.417 |
| Office hypertension | 0.161 | 0.103 to 0.218 |
| Isolated nocturnal hypertension | 0.241 | 0.185 to 0.296 |
| Non dipper | 0.265 | 0.208 to 0.319 |
| Extreme dipper | 0.178 | 0.120 to 0.234 |
| Reverse dipper | 0.0712 | 0.0121 to 0.129 |

**Table S4.** Correlation coefficients (C) and p-values (p) for office and ambulatory blood pressures measured at baseline (1) and repeat (2) assessment.

**SYSTOLIC BLOOD PRESSURE**

| **Variable** | Office-1 | | Office-2 | 24-hour-1 | 24-hour-2 | Day-1 | Day-2 | Night-1 | Night-2 |
| --- | --- | --- | --- | --- | --- | --- | --- | --- | --- |
| Office-1 |  | 1 |  |  |  |  |  |  |  |
| Office-2 | Cp | 0.460 <0.0001 | 1 |  |  |  |  |  |  |
| 24-hour-1 | Cp | 0.273 <0.0001 | 0.333 <0.0001 | 1 |  |  |  |  |  |
| 24-hour-2 | Cp | 0.173 <0.0001 | 0.352 <0.0001 | 0.727* <0.0001 | 1 |  |  |  |  |
| Day-1 | Cp | 0.274 <0.0001 | 0.337 <0.0001 | 0.983 <0.0001 | 0.715 <0.0001 | 1 |  |  |  |
| Day-2 | Cp | 0.168 <0.0001 | 0.351 <0.0001 | 0.708 <0.0001 | 0.982 <0.0001 | 0.711*† <0.0001 | 1 |  |  |
| Night-1 | Cp | 0.189 <0.0001 | 0.265 <0.0001 | 0.805 <0.0001 | 0.610 <0.0001 | 0.712 <0.0001 | 0.565 <0.0001 | 1 |  |
| Night-2 | Cp | 0.135 <0.0001 | 0.294 <0.0001 | 0.600 <0.0001 | 0.815 <0.0001 | 0.564 <0.0001 | 0.724 <0.0001 | 0.634 <0.0001 | 1 |

*p≤0.001 versus night-time correlation coefficient. †p=N.S. versus 24-hour correlation coefficient

**DIASTOLIC BLOOD PRESSURE**

| **Variable** | Office-1 | | Office-2 | | 24-hour-1 | 24-hour-2 | | Day-1 | | Day-2 | | Night-1 | | Night-2 | |  |
| --- | --- | --- | --- | --- | --- | --- | --- | --- | --- | --- | --- | --- | --- | --- | --- | --- |
| Office-1 |  | 1 | |  |  | |  | |  | |  | |  | |  | |
| Office-2 | Cp | 0.446 <0.0001 | | 1 |  | |  | |  | |  | |  | |  | |
| 24-hour-1 | Cp | 0.375 <0.0001 | | 0.344 <0.0001 | 1 | |  | |  | |  | |  | |  | |
| 24-hour-2 | Cp | 0.308 <0.0001 | | 0.382 <0.0001 | 0.709* <0.0001 | | 1 | |  | |  | |  | |  | |
| Day-1 | Cp | 0.358 <0.0001 | | 0.334 <0.0001 | 0.982 <0.0001 | | 0.697 <0.0001 | | 1 | |  | |  | |  | |
| Day-2 | Cp | 0.280 <0.0001 | | 0.368 <0.0001 | 0.696 <0.0001 | | 0.982 <0.0001 | | 0.703*† <0.0001 | | 1 | |  | |  | |
| Night-1 | Cp | 0.319 <0.0001 | | 0.300 <0.0001 | 0.810 <0.0001 | | 0.585 <0.0001 | | 0.709 <0.0001 | | 0.530 <0.0001 | | 1 | |  | |
| Night-2 | Cp | 0.266 <0.0001 | | 0.324 <0.0001 | 0.552 <0.0001 | | 0.783 <0.0001 | | 0.499 <0.0001 | | 0.679 <0.0001 | | 0.609 <0.0001 | | 1 | |

*p<0.001 versus night-time correlation coefficient. . †p=N.S. versus 24-hour correlation coefficient.
